# Cyclo­hexane-1,2,3,4,5-pentol

**DOI:** 10.1107/S1600536809014135

**Published:** 2009-04-25

**Authors:** G. Ganesh, C. Sivaraj, P. S. Kannan, N. Raaman, A. SubbiahPandi

**Affiliations:** a Department of Physics, SMK Fomra Institute of Technology, Thaiyur, Chennai 603 103, India; b CAS in Botany, University of Madras, Guindy Campus, Chennai 600 025, India; c Department of Physics, Presidency College (Autonomous), Chennai 600 005, India

## Abstract

In the title compound, C_6_H_12_O_5_, the cyclo­hexane ring adopts a chair conformation. The absolute configuration is not defined. However, the relative configuration can be assigned as 1R*,3R*,4S*,S*. In the crystal structure, mol­ecules are linked by strong inter­molecular O—H⋯O hydrogen bonds, producing a three-dimensional network.

## Related literature

For details of the biological activity and applications of cyclohexane derivatives, see: Eddington *et al.* (2000 ▶); Padmavathi *et al.* (2000 ▶, 2001 ▶); Li & Strobel (2001 ▶). For puckering parameters and displacement asymmetric parameters, see: Cremer & Pople (1975 ▶); Nardelli (1983 ▶).
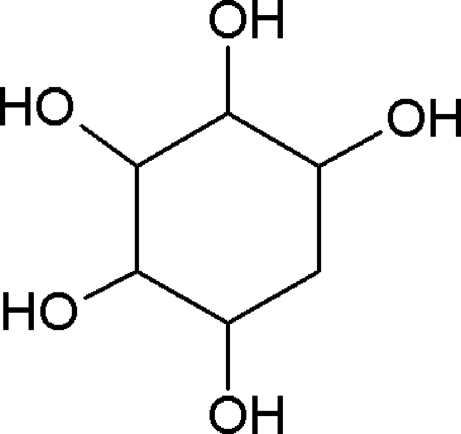


## Experimental

### 

#### Crystal data

C_6_H_12_O_5_
*M* *_r_* = 164.16
Monoclinic, 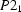

*a* = 6.4727 (5) Å
*b* = 8.4851 (6) Å
*c* = 6.8249 (5) Å
β = 110.796 (2)°
*V* = 350.41 (5) Å^3^
*Z* = 2
Mo *K*α radiation
μ = 0.14 mm^−1^
*T* = 293 K
0.21 × 0.19 × 0.17 mm

#### Data collection

Bruker Kappa APEXII CCD diffractometer
Absorption correction: multi-scan (*SADABS*; Sheldrick, 1996 ▶) *T*_min_ = 0.972, *T*_max_ = 0.977
5019 measured reflections
2418 independent reflections
2314 reflections with *I* > 2σ(*I*)
*R*_int_ = 0.020

#### Refinement

*R*[*F* ^2^ > 2σ(*F* ^2^)] = 0.033
*wR*(*F* ^2^) = 0.086
*S* = 1.06
2418 reflections
105 parameters
1 restraint
H-atom parameters constrained
Δρ_max_ = 0.39 e Å^−3^
Δρ_min_ = −0.19 e Å^−3^
Absolute structure: Flack (1983 ▶), 994 Friedel pairs
Flack parameter: 0.7 (6)

### 

Data collection: *APEX2* (Bruker, 2004 ▶); cell refinement: *APEX2*; data reduction: *SAINT* (Bruker, 2004 ▶); program(s) used to solve structure: *SHELXS97* (Sheldrick, 2008 ▶); program(s) used to refine structure: *SHELXL97* (Sheldrick, 2008 ▶); molecular graphics: *ORTEP-3* (Farrugia, 1997 ▶); software used to prepare material for publication: *SHELXL97* and *PLATON* (Spek, 2009 ▶).

## Supplementary Material

Crystal structure: contains datablocks global, I. DOI: 10.1107/S1600536809014135/kp2211sup1.cif

Structure factors: contains datablocks I. DOI: 10.1107/S1600536809014135/kp2211Isup2.hkl

Additional supplementary materials:  crystallographic information; 3D view; checkCIF report

## Figures and Tables

**Table 1:** Hydrogen-bond geometry (Å, °)

*D*—H⋯*A*	*D*—H	H⋯*A*	*D*⋯*A*	*D*—H⋯*A*
O1—H1*A*⋯O3^i^	0.82	1.94	2.7347 (11)	164
O2—H2*A*⋯O4^ii^	0.82	1.96	2.7761 (12)	170
O3—H3*A*⋯O1^iii^	0.82	2.02	2.8417 (11)	177
O4—H4*A*⋯O5^iv^	0.82	1.91	2.7067 (12)	165
O5—H5⋯O2^v^	0.82	2.00	2.8036 (12)	166

Symmetry codes: (i) 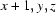; (ii) 
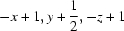
; (iii) 
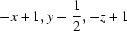
; (iv) 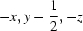; (v) 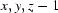.

**Fig. 1.**
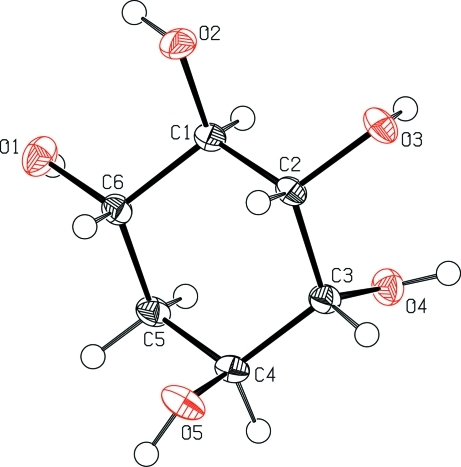
The molecular structure of the title compound showing the 50% probability displacement ellipsoids.

**Fig. 2.**
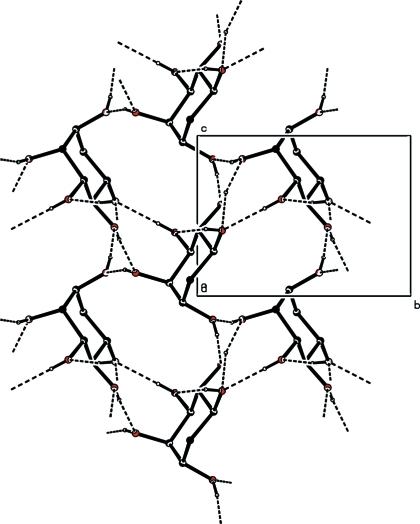
The packing diagram of the title compound, view along the a axis forming a three dimensional network.

## supplementary crystallographic information

### Comment

Cyclohexanes are either prepared from natural sources or entirely *via* synthetic routes. The reason for their preparation is a variety of medical effects. The molecules provide anticonvulsant, antimalarial, antiinflamatory and cardiovascular effects (Eddington *et al.*, 2000). Cyclohexanes are also important intermediates for many biologically active compounds (Padmavathi *et al.*, 2001; Padmavathi *et al.*, 2000). A number of their derivatives have fungicidal and antitumor activities (Li & Strobel, 2001). Taking into consideration of these aspects, and in order to obtain a detailed information on the molecular structure in the solid state, the X-ray study of the title compound has been carried out.

X-Ray analysis confirms the molecular structure and atom connectivity for (I) (Fig. 1).The cyclohexane ring adopts the chair conformation with the puckering parameters q_2_ and φ (Cremer & Pople, 1975) and the smallest displacement asymmetric parameters, Δ, (Nardelli, 1983) as follows: q_2_=0.0673 (11) Å, φ=111.3 (9)°, Δ_s_(C1)= 1.27 (8).

The atom O1 acts as a donor to the atom O3 of the neighbouring molecule. This hydrogen bond is involved in a motif C(6) forming an infinite chain along *a* axis, and also the atom O5 acts as a donor to the atom O2. This hydrogen bond is involved in a motif C(7) forming an infinite chain along *c* axis. The crystal packing is defined by O–H···O hydrogen bonds (Table 1, Fig. 2)

### Experimental

The compound was isolated from Manilkara zapota(*L*) Van Royan leaves of ethyl acetate fraction by column chromatography. Single crystals of the title compound suitable for X-ray diffraction were obtained from a mixture of ethyl acetate and methanol (3:1) by slow evaporation.

### Refinement

All the H atoms were positioned geometrically, with O—H = 0.82 Å and C—H = 0.93 - 0.98 Å and constrained to ride on their parent atom, with *U*_iso_H=1.2*U*_eq_(C).

### Crystal data

**Table:**

C_6_H_12_O_5_	*F*(000) = 176
*M**_r_* = 164.16	*D*_x_ = 1.556 Mg m^−^^3^
Monoclinic, *P*2_1_	Mo *K*α radiation, λ = 0.71073 Å
Hall symbol: P 2yb	Cell parameters from 2418 reflections
*a* = 6.4727 (5) Å	θ = 3.2–33.3°
*b* = 8.4851 (6) Å	µ = 0.14 mm^−^^1^
*c* = 6.8249 (5) Å	*T* = 293 K
β = 110.796 (2)°	Block, colourless
*V* = 350.41 (5) Å^3^	0.21 × 0.19 × 0.17 mm
*Z* = 2	

### Data collection

**Table:**

Bruker Kappa APEXII CCD diffractometer	2418 independent reflections
Radiation source: fine-focus sealed tube	2314 reflections with *I* > 2σ(*I*)
graphite	*R*_int_ = 0.020
ω scans	θ_max_ = 33.3°, θ_min_ = 3.2°
Absorption correction: multi-scan (*SADABS*; Sheldrick, 1996)	*h* = −9→9
*T*_min_ = 0.972, *T*_max_ = 0.977	*k* = −12→12
5019 measured reflections	*l* = −9→10

### Refinement

**Table:**

Refinement on *F*^2^	Secondary atom site location: difference Fourier map
Least-squares matrix: full	Hydrogen site location: inferred from neighbouring sites
*R*[*F*^2^ > 2σ(*F*^2^)] = 0.033	H-atom parameters constrained
*wR*(*F*^2^) = 0.086	*w* = 1/[σ^2^(*F*_o_^2^) + (0.0548*P*)^2^ + 0.0106*P*] where *P* = (*F*_o_^2^ + 2*F*_c_^2^)/3
*S* = 1.06	(Δ/σ)_max_ < 0.001
2418 reflections	Δρ_max_ = 0.39 e Å^−^^3^
105 parameters	Δρ_min_ = −0.19 e Å^−^^3^
1 restraint	Absolute structure: Flack (1983), 994 Friedel pairs
Primary atom site location: structure-invariant direct methods	Flack parameter: 0.7 (6)

### Special details

**Table:**

Geometry. All e.s.d.'s (except the e.s.d. in the dihedral angle between two l.s. planes) are estimated using the full covariance matrix. The cell e.s.d.'s are taken into account individually in the estimation of e.s.d.'s in distances, angles and torsion angles; correlations between e.s.d.'s in cell parameters are only used when they are defined by crystal symmetry. An approximate (isotropic) treatment of cell e.s.d.'s is used for estimating e.s.d.'s involving l.s. planes.
Refinement. Refinement of *F*^2^ against ALL reflections. The weighted *R*-factor *wR* and goodness of fit *S* are based on *F*^2^, conventional *R*-factors *R* are based on *F*, with *F* set to zero for negative *F*^2^. The threshold expression of *F*^2^ > σ(*F*^2^) is used only for calculating *R*-factors(gt) *etc*. and is not relevant to the choice of reflections for refinement. *R*-factors based on *F*^2^ are statistically about twice as large as those based on *F*, and *R*- factors based on ALL data will be even larger.

### Fractional atomic coordinates and isotropic or equivalent isotropic displacement parameters (Å^2^)

**Table:**

	*x*	*y*	*z*	*U*_iso_*/*U*_eq_	
C1	0.39464 (15)	0.00491 (11)	0.41069 (16)	0.01595 (17)	
H1	0.4688	−0.0926	0.4757	0.019*	
C2	0.15563 (14)	−0.03135 (11)	0.27557 (15)	0.01522 (17)	
H2	0.0821	0.0694	0.2247	0.018*	
C3	0.13572 (14)	−0.13189 (11)	0.08297 (15)	0.01626 (17)	
H3	−0.0198	−0.1352	−0.0093	0.020*	
C4	0.27248 (16)	−0.06837 (11)	−0.04069 (15)	0.01663 (17)	
H4	0.2741	−0.1468	−0.1456	0.020*	
C5	0.51009 (15)	−0.03378 (11)	0.10101 (16)	0.01768 (18)	
H5A	0.5837	−0.1318	0.1590	0.021*	
H5B	0.5889	0.0137	0.0188	0.021*	
C6	0.51703 (15)	0.07685 (11)	0.27875 (16)	0.01628 (17)	
H6	0.4414	0.1747	0.2168	0.020*	
O1	0.73862 (11)	0.11569 (9)	0.40896 (14)	0.02328 (17)	
H1A	0.8190	0.0392	0.4180	0.035*	
O2	0.38759 (13)	0.11155 (10)	0.57023 (13)	0.02410 (17)	
H2A	0.5140	0.1337	0.6464	0.036*	
O3	0.04224 (12)	−0.10449 (9)	0.39647 (13)	0.02046 (16)	
H3A	0.1094	−0.1839	0.4529	0.031*	
O4	0.20891 (12)	−0.28909 (9)	0.14488 (13)	0.02034 (16)	
H4A	0.1094	−0.3390	0.1642	0.031*	
O5	0.16705 (13)	0.07199 (9)	−0.14612 (13)	0.02343 (17)	
H5	0.2119	0.0922	−0.2416	0.035*	

### Atomic displacement parameters (Å^2^)

**Table:**

	*U*^11^	*U*^22^	*U*^33^	*U*^12^	*U*^13^	*U*^23^
C1	0.0173 (4)	0.0179 (4)	0.0146 (4)	−0.0003 (3)	0.0080 (3)	−0.0012 (3)
C2	0.0156 (3)	0.0175 (4)	0.0152 (4)	0.0008 (3)	0.0088 (3)	0.0005 (3)
C3	0.0166 (3)	0.0182 (4)	0.0147 (4)	0.0003 (3)	0.0065 (3)	−0.0001 (3)
C4	0.0192 (4)	0.0187 (4)	0.0136 (4)	0.0020 (3)	0.0078 (3)	0.0000 (3)
C5	0.0179 (4)	0.0215 (4)	0.0167 (4)	0.0012 (3)	0.0099 (3)	−0.0006 (3)
C6	0.0159 (3)	0.0180 (4)	0.0173 (4)	−0.0007 (3)	0.0088 (3)	−0.0006 (3)
O1	0.0168 (3)	0.0249 (3)	0.0288 (4)	−0.0031 (3)	0.0089 (3)	−0.0074 (3)
O2	0.0253 (3)	0.0310 (4)	0.0205 (4)	−0.0075 (3)	0.0136 (3)	−0.0112 (3)
O3	0.0215 (3)	0.0226 (3)	0.0233 (4)	0.0010 (3)	0.0155 (3)	0.0027 (3)
O4	0.0231 (3)	0.0162 (3)	0.0237 (4)	−0.0008 (2)	0.0107 (3)	0.0000 (3)
O5	0.0265 (3)	0.0278 (4)	0.0195 (4)	0.0093 (3)	0.0125 (3)	0.0094 (3)

### Geometric parameters (Å, °)

**Table:**

C1—O2	1.4289 (13)	C4—H4	0.9800
C1—C6	1.5223 (14)	C5—C6	1.5218 (14)
C1—C2	1.5249 (12)	C5—H5A	0.9700
C1—H1	0.9800	C5—H5B	0.9700
C2—O3	1.4264 (12)	C6—O1	1.4319 (11)
C2—C3	1.5337 (14)	C6—H6	0.9800
C2—H2	0.9800	O1—H1A	0.8200
C3—O4	1.4282 (12)	O2—H2A	0.8200
C3—C4	1.5221 (14)	O3—H3A	0.8200
C3—H3	0.9800	O4—H4A	0.8200
C4—O5	1.4328 (12)	O5—H5	0.8200
C4—C5	1.5266 (13)		
			
O2—C1—C6	111.16 (8)	O5—C4—H4	108.9
O2—C1—C2	106.77 (7)	C3—C4—H4	108.9
C6—C1—C2	110.64 (8)	C5—C4—H4	108.9
O2—C1—H1	109.4	C6—C5—C4	111.20 (8)
C6—C1—H1	109.4	C6—C5—H5A	109.4
C2—C1—H1	109.4	C4—C5—H5A	109.4
O3—C2—C1	110.97 (8)	C6—C5—H5B	109.4
O3—C2—C3	110.48 (7)	C4—C5—H5B	109.4
C1—C2—C3	112.89 (7)	H5A—C5—H5B	108.0
O3—C2—H2	107.4	O1—C6—C5	112.10 (8)
C1—C2—H2	107.4	O1—C6—C1	110.01 (8)
C3—C2—H2	107.4	C5—C6—C1	110.48 (8)
O4—C3—C4	107.32 (8)	O1—C6—H6	108.0
O4—C3—C2	110.35 (8)	C5—C6—H6	108.0
C4—C3—C2	112.94 (7)	C1—C6—H6	108.0
O4—C3—H3	108.7	C6—O1—H1A	109.5
C4—C3—H3	108.7	C1—O2—H2A	109.5
C2—C3—H3	108.7	C2—O3—H3A	109.5
O5—C4—C3	107.80 (8)	C3—O4—H4A	109.5
O5—C4—C5	110.57 (8)	C4—O5—H5	109.5
C3—C4—C5	111.73 (8)		
			
O2—C1—C2—O3	−61.20 (10)	O4—C3—C4—C5	−72.15 (10)
C6—C1—C2—O3	177.71 (7)	C2—C3—C4—C5	49.69 (10)
O2—C1—C2—C3	174.15 (8)	O5—C4—C5—C6	65.25 (11)
C6—C1—C2—C3	53.07 (10)	C3—C4—C5—C6	−54.82 (11)
O3—C2—C3—O4	−54.15 (10)	C4—C5—C6—O1	−177.75 (8)
C1—C2—C3—O4	70.77 (10)	C4—C5—C6—C1	59.17 (10)
O3—C2—C3—C4	−174.27 (7)	O2—C1—C6—O1	59.39 (10)
C1—C2—C3—C4	−49.35 (10)	C2—C1—C6—O1	177.84 (8)
O4—C3—C4—O5	166.16 (7)	O2—C1—C6—C5	−176.32 (7)
C2—C3—C4—O5	−71.99 (10)	C2—C1—C6—C5	−57.87 (9)

### Hydrogen-bond geometry (Å, °)

**Table:**

*D*—H···*A*	*D*—H	H···*A*	*D*···*A*	*D*—H···*A*
O1—H1A···O3^i^	0.82	1.94	2.7347 (11)	164
O2—H2A···O4^ii^	0.82	1.96	2.7761 (12)	170
O3—H3A···O1^iii^	0.82	2.02	2.8417 (11)	177
O4—H4A···O5^iv^	0.82	1.91	2.7067 (12)	165
O5—H5···O2^v^	0.82	2.00	2.8036 (12)	166

Symmetry codes: (i) *x*+1, *y*, *z*; (ii) −*x*+1, *y*+1/2, −*z*+1; (iii) −*x*+1, *y*−1/2, −*z*+1; (iv) −*x*, *y*−1/2, −*z*; (v) *x*, *y*, *z*−1.
